# Effect of Indirect Nonequilibrium Atmospheric Pressure Plasma on Anti-Proliferative Activity against Chronic Chemo-Resistant Ovarian Cancer Cells *In Vitro* and *In Vivo*

**DOI:** 10.1371/journal.pone.0081576

**Published:** 2013-12-18

**Authors:** Fumi Utsumi, Hiroaki Kajiyama, Kae Nakamura, Hiromasa Tanaka, Masaaki Mizuno, Kenji Ishikawa, Hiroki Kondo, Hiroyuki Kano, Masaru Hori, Fumitaka Kikkawa

**Affiliations:** 1 Department of Obstetrics and Gynecology, Nagoya University Graduate School of Medicine, Showa-ku, Nagoya, Japan; 2 Department of Electrical Engineering and Computer Science, Graduate School of Engineering, Nagoya University, Chikusa-ku, Nagoya, Japan; 3 Center for Advanced Medicine and Clinical Research, Nagoya University Graduate School of Medicine, Showa-ku, Nagoya, Japan; 4 NU Eco-Engineering Co., Ltd., Miyoshi-shi, Aichi, Japan; University Paul Sabatier, France

## Abstract

**Purpose:**

Nonequilibrium atmospheric pressure plasma (NEAPP) therapy has recently been focused on as a novel medical practice. Using cells with acquired paclitaxel/cisplatin resistance, we elucidated effects of indirect NEAPP-activated medium (NEAPP-AM) exposure on cell viability and tumor growth *in vitr*o and *in vivo*.

**Methods:**

Using chronic paclitaxel/cisplatin-resistant ovarian cancer cells, we applied indirect NEAPP-exposed medium to cells and *xenografted* tumors in a mouse model. Furthermore, we examined the role of reactive oxygen species (ROS) or their scavengers in the above-mentioned EOC cells.

**Results:**

We assessed the viability of NOS2 and NOS3 cells exposed to NEAPP-AM, which was prepared beforehand by irradiation with NEAPP for the indicated time. In NOS2 cells, viability decreased by approximately 30% after NEAPP-AM 120-sec treatment (*P*<0.01). The growth-inhibitory effects of NEAPP-AM were completely inhibited by N-acetyl cysteine treatment, while L-buthionine-[S, R]-sulfoximine, an inhibitor of the ROS scavenger used with NEAPP-AM, decreased cell viability by 85% after NEAPP-AM 60-sec treatment(*P*<0.05) and by 52% after 120 sec, compared to the control (*P*<0.01). In the murine subcutaneous tumor-formation model, NEAPP-AM injection resulted in an average inhibition of the NOS2 cell-inoculated tumor by 66% (*P*<0.05) and NOS2TR cell-inoculated tumor by 52% (*P*<0.05), as compared with the control.

**Conclusion:**

We demonstrated that plasma-activated medium also had an anti-tumor effect on chemo-resistant cells *in vitro* and *in vivo*. Indirect plasma therapy is a promising treatment option for EOC and may contribute to a better patient prognosis in the future.

## Introduction

Epithelial ovarian carcinoma (EOC) is the fifth leading cause of cancer-related death and remains one of the most aggressive tumors of all gynecologic malignancies in Western countries. According to Cancer Statistics 2012, it was estimated that 15,500 died of the disease in the United States [1]. The majority of patients with EOC have advanced intraperitoneal metastatic disease at diagnosis since this carcinoma frequently remains clinically silent. Since the treatment strategy consisting of maximum cytoreductive surgery followed by taxane plus platinum chemotherapy was established, the short-term prognosis of patients with EOC has improved. However, despite the comparatively high-level sensitivity of EOC to paclitaxel, the prognosis of advanced or recurrent cases remains poor because most mortality cases are the result of metastasis that is refractory to these chemotherapeutic agents. Although various additional molecular-targeting therapies, including anti-angiogenic agents, have been investigated in order to overcome such paclitaxel resistance, the effect of such treatment is not satisfactory [2], [3].

Plasma, commonly employed in physical sciences, is essentially an ionized gas in which a fraction of the atoms or molecules is ionized [4]. Plasma as an active ionized medium sustained by the supply of energy containing free charges, free radicals, excited molecules and energetic photons can induce processes usually obtained through chemical treatment or radiotherapy [5]. Due to technical developments, new-generation plasma called nonequilibrium atmospheric pressure plasma (NEAPP), also known as cold plasma or non-thermal plasma, has actually entered practical use [6]. Recently, NEAPP therapy has been focused on as a novel medical practice [7]–[9]. In early plasma use in life-science, thermal plasmas were utilized for surgical tool and sterilization. In recent plasma applications, researchers mainly focus on the non-thermal plasma effects involving its reactive species (RS) for biological objects. There have been therapeutic trials applied in the fields of tissue sterilization, blood coagulation, wound-healing promotion, and dental bleaching [10]–[12]. Additionally, it was recently reported that plasma exerted anti-proliferative effects on a variety of cancer cells, inducing apoptosis, which is known as programmed cell death [5], [13], [14]. According to the examination from Sensenig et al., NEAPP treatment induces apoptosis through a pathway that appears to be dependent on the production of intracellular ROS, leading to dose-dependent DNA damage in melanoma cells [14]. Moreover, instead of a direct effect on the cells, exposure to medium treated with plasma separated from cells (indirect plasma) led to a reduction in the cell proliferation of mammalian breast epithelial cells and HeLa cells via the generation of ROS in the medium [13], [15]. It was noteworthy that in the dielectric barrier plasma, a type of non-thermal plasma used as an indirect plasma source, filamentary discharges contacted directly with the medium as a counter electrode against an insulating electrode powered during treatments. Therefore the precise roles of ions and radicals generated electric discharges are unresolved.

On the other hand, selective targeting of tumor cells apart from surrounding normal cells is crucial for any anti-cancer therapeutic strategies. In our recent report, two independent human EOC cell lines and normal fibroblast cells treated with high-flux NEAPP were evaluated by toxicity and proliferation assays. As a result, both types of EOC cell were discriminately killed through inducing enhanced apoptosis, while plasma-treated fibroblast cells were not damaged [16]. More recently, we also demonstrated that glioblastoma cells were selectively induced to undergo apoptosis when treated with indirect plasma-exposed medium through AKT down-regulation [17]. In the present study, using cells with acquired paclitaxel/cisplatin resistance, established previously [18], [19], we elucidated the effect of indirect NEAPP-activated medium (NEAPP-AM) exposure on cell viability and tumor growth *in vitr*o and *in vivo*. Furthermore, we examined the role of reactive oxygen species (ROS) or their scavengers in chronic antineoplastic-resistant EOC cells. A possible application of this technologic modality as a therapeutic target for EOC is proposed.

## Materials and Methods

### Cell culture

The NOS2 and NOS3 (also known as; TAOV) cells, derived from serous EOC, were established in our institute [20], [21]. These cell lines were maintained in RPMI-1640 (Sigma, St. Louis, MO, USA) supplemented with 10% fetal Bovine serum (FBS) and penicillin-streptomycin at 37°C in a humidified atmosphere of 5% CO_2_.

### Establishment of paclitaxel/cisplatin-resistant EOC cells

The NOS2TR and NOS2CR cells, established from parental NOS2 cells in our institute [18], [19], had acquired chronic resistance to paclitaxel and cisplatin, respectively. In addition to these lines, we newly generated another two chronic paclitaxel/cisplatin-resistant lines from parental NOS3 cells: NOS3TR (paclitaxel) and NOS3CR (cisplatin). Briefly, NOS3 cells were washed thoroughly with PBS, and transferred to RPMI-1640 medium containing 10% FBS and penicillin-streptomycin. The cells were continuously exposed to paclitaxel (Bristol Myers squib, Tokyo, Japan) or cisplatin (Nihon Kayaku, Tokyo, Japan) for more than 6 months, during which time the medium was replaced every 3–4 days and the cell cultures were passaged by trypsinization after subconfluency was reached. Gradually, these cells displayed resistance to the growth-inhibitory properties of paclitaxel or cisplatin. These cell lines, designated paclitaxel or cisplatin-resistant, were cultured for a further 3 months in medium containing these antineoplastic agents before characterization studies. Through these processes, we finally generated four paclitaxel/cisplatin-resistant EOC cell lines {NOS2TR and NOS3TR (paclitaxel), NOS2CR and NOS3CR (cisplatin)}. These cell lines were maintained in RPMI-1640 supplemented with 10% FBS and penicillin-streptomycin at 37°C in a humidified atmosphere of 5% CO_2_.

### Experimental system specification and production of nonequilibrium atmospheric pressure plasma-activated medium (NEAPP-AM)

**Figure 1:**
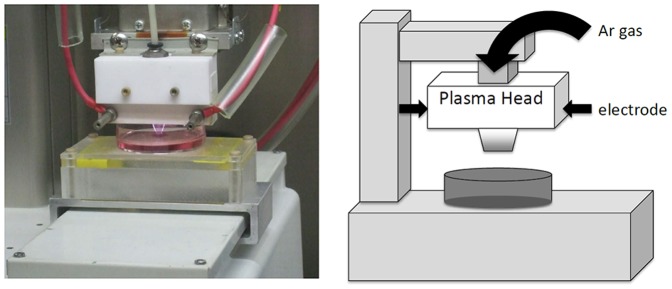
Scheme of generation of nonequilibrium atmospheric pressure plasma (NEAPP)-activated medium.

The details of this experimental NEAPP system, as shown in Fig. 1, were previously described. Discharge conditions were in argon gas (2 standard liters/min; slm) excited by applying 10 kV of a 60-Hz commercial power supply to two electrodes with a distance of 8 mm [16]. In brief, NEAPP with an ultra-high electron density (approximately 2×10^16^ cm^−3^) provided characteristically with an ultra-high O density estimated of around 4×10^15^ cm^−3^ [22], [23]. Furthermore, generation of reactive oxygen species (ROS), such as hydroxyl radicals, singlet oxygen radicals, nitrogen oxide, and nitrogen, were confirmed by optical emission spectroscopy (OES). We exposed above NEAPP to RPMI-1640 separately from the cells, which is designated ‘Nonequilibrium atmospheric pressure plasmas activated medium (NEAPP-AM)’. The separated distance between the plasma source and medium (L) is critical to consistently reproduce data, and so all experiments were performed under the same conditions, L = 15 mm, where no plasma discharge were contacted with the medium. The duration of plasma treatment was ranged from 0 to 300 seconds *in vitro* studies. Six mL of RPMI-1640 medium was placed in 60-mm dish. The center on each 60-mm dish was treated for several exposure times (30, 60, 120, 180, and 300 sec) with NEAPP, indicated by NEAPP-AM-30, -60, -120, -180 and -300 respectively below. For animal treatment, four mL of medium was placed in 21-mm dish and was treated with NEAPP for 600 sec.

### Chemosensitivity assay

The paclitaxel/cisplatin chemo-sensitivity assay was performed as described previously [24]. Briefly, cells were seeded in triplicate in 96-well plates at a density of 2,000 cells in a volume of 100 µL of culture medium containing 10% FBS. After incubation for 24 hrs at 37°C, the medium was replaced with fresh medium with or without various concentrations of paclitaxel and cisplatin. After an additional 72 hr, cell viability was assayed using the Aqueous One Solution Cell Proliferation Assay kit (Promega, Madison, WI, USA), according to the manufacturer's instructions. Absorbance was then measured at 490 nm with a microplate reader (Multiskan Bichromatic; Labsystems, Helsinki, Finland). IC50 values indicate the concentrations resulting in a 50% reduction in growth as compared with control cell growth.

### Cell viability assay

The effect of NEAPP-AM on the viability of cells was determined by the Aqueous One Solution Cell Proliferation Assay kit (Promega, Madison, WI, USA) described in “Chemosensitivity assay”. The cells were plated in 96-well plates at a density of 1×10^4^ cells per well in 100 µL of complete culture medium. The next day, cells were treated with NEAPP-AM (30–300 sec/6 mL) for 24 hrs, and the above conditions were optimized to detect the NEAPP-AM sensitivity of the cells. Each activated time for NEAPP-AM was repeated in 6 wells. Experiments were performed in triplicate.

### Reactive oxidative species (ROS) inhibition and L-γ-glutamyl-L-cysteinyl-glycine (GSH) depletion

To inhibit ROS, N-acetyl cysteine (NAC, Sigma-Ardrich, St. Louis, MO, USA), an intracellular ROS scavenger, was used. In addition, L-buthionine-[S, R]-sulfoximine (BSO, Sigma-Ardrich, St. Louis, MO, USA) is an inhibitor of GSH synthesis. It is known that GSH is the most abundant and effective component of the defense system against free radicals including ROS. The compounds NAC and BSO were added to cells at a final concentration of 4 and 2 mM in PBS, respectively. The required volume of each drug was added directly to complete cell culture medium 2 hrs before NEAPP-AM treatment and NEAPP-AM to achieve the desired final concentrations, respectively. Cell viability was examined with the “Cell viability assay”.

### Cell apoptosis assay/caspase-3/7 activity assay

The activity of caspase-3/7 was determined with the CellEvent™ caspase-3/7 Green Detection Reagent (Molecular Probes Invitrogen, Calsbad, CA) according to the manufacturer's instructions. NOS2 and NOS2TR cells (1.5×10^4^/well) were seeded in an 8-well imaging chamber (Lab-Tek Thermo Fisher Scientific Inc., Waltham, MA), incubated for 24 hrs, and then treated with NEAPP-AM or serum free medium as a control. After 2 hrs of incubation, CellEvent™ caspase-3/7 Green Detection Reagent was added to the wells at a final concentration of 10 µM. Four hrs after NEAPP-AM treatment, cells were observed with a light and a fluorescence microscope. This experiment was repeated at least three times.

### Detection of intracellular ROS accumulation

Intracellular ROS accumulation was monitored using 5–6-chloromethyl-2′7′-dichlorodihydroflorescein diacetate, acetyl ester (CM-H_2_DCFDA; Molecular Probes Invitrogen, Calsbad, CA). To detect the cellular ROS level, CM-H_2_DCFDA (4 µM) in PBS was loaded for 15 minutes at 37°C in the dark. After loading, buffer was changed to culture media or NEAPP-AM, and cells were incubated for 30 min at 37°C, and observed by fluorescence microscopy. The production of ROS can be visualized by changes in fluorescence due to the intracellular production of CM-DCF caused by the oxidation of CM-H_2_DCF.

### Animal studies

A total of 1×10^3^ NOS2 and NOS2TR cells were suspended in 150 µL of serum free medium and 150 µL of Matrigel (BD Biosciences, San Jose, CA, USA), and used to subcutaneously inoculate both sides of the flank of 8-week-old female nude mice (BALB/C) (N = 12) (Japan SLC, Nagoya, Japan) using a 27-gauge needle, and they were then randomly divided into two equal groups, respectively. This animal experiment protocol was approved by the Animal Experimental Committee of the Graduate School of Medicine, Nagoya University.

One group of mice received 200 µL of NEAPP-AM by subcutaneous injection in each side, and the other group received the same volume of non-plasma exposed RPMI-1640 medium as a control group. The NEAPP-AM was prepared as described above. These treatments were repeated 3 times a week, basically on Mondays, Wednesdays, and Fridays, from 24 hrs after cell injection. Once a tumor started growing, it was measured with calipers and the volume was calculated using the formula: π/6×(largest diameter)×(smallest diameter)^2^. At the termination of the experiment (29 days after implantation), mice were sacrificed and tumor tissues were harvested and weighed. Hematoxylin-eosin (H&E) staining was performed of sections from paraffin-embedded tumors. Morphological differences were assessed by microscopy of the stained sections in both groups.

### Statistical analysis

Data are presented as means ± SD from at least three independent experiments. Statistical analysis of the data was performed using the Student's *t*-test. Differences between groups were considered significant at *P*<0.05.

## Results

### Effect of NEAPP-AM on cell growth and viability

**Figure 2:**
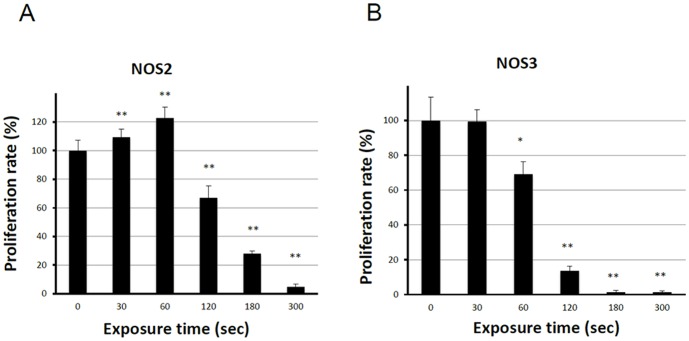
Effect of plasma on cell viability. Viability of NOS2 and NOS3 cells treated with NEAPP-activated medium (NEAPP-AM) as measured by the cell viability assay. NOS2 (A) and NOS3 (B) human ovarian cancer cells were plated in 96-well plates and incubated at 37°C with 5% CO2. After 24 hrs, the culture medium was replaced with NEAPP-AM and the cells were incubated for another 24 hrs. The percentages of surviving cells from each cell line were calculated relative to controls. The plasma exposure time for zero was determined with the cell viability assay. Each column represents the mean, and the bars show SD. Data are representative of three independent experiments. *P<0.05, **P<0.01 versus control.

We first evaluated the anti-tumor potential of NEAPP-AM on the growth of two different human EOC cell lines *in vitro* using the cell viability assay. Figure 2 A–B shows the cell viability of NOS2 and NOS3 cells exposed to NEAPP-AM for 24 hrs, which was prepared beforehand by irradiation with NEAPP for the indicated time. In NOS2 cells, the viability decreased by approximately 30% when cells were treated with NEAPP-AM-120, and 70% after being treated with NEAPP-AM-180(*P*<0.01) compared to non-NEAPP-exposed medium treatment. A similar tendency was also shown in NOS3 cells, in which cell proliferation was down-regulated by approximately 30% after being treated with NEAPP-AM-60(*P*<0.05), and 90% after treatment with NEAPP-AM-120 (*P*<0.01). In contrast, on exposure for a shorter duration, cell proliferation was up-regulated with an approximately 20% increase.

### Role of ROS and enhanced NEAPP-AM sensitivity with BSO

**Figure 3:**
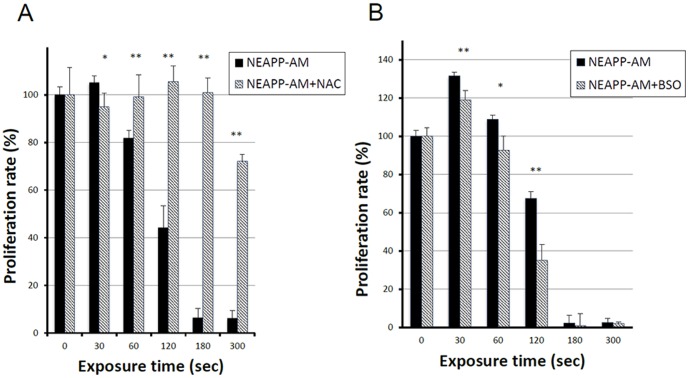
Role of ROS in NEAPP-AM. *A, B:* Influence of intracellular ROS modulation by NAC and BSO on NEAPP-AM-induced cell death. A: NOS2 cells were pretreated with NAC (4 mM)(A) or BSO (2 m M) (B) for 2 hrs and then exposed to NEAPP-AM with NAC or BSO for an additional 24 hrs. The cell viability assay was used for evaluation. Each column represents the mean and the bars are SD. Data are representative of at least three independent experiments. *P<0.05, **P<0.01 versus control without NAC treatment.

Subsequently, we examined whether ROS produced by NEAPP have anti-tumor effects. To test the role of ROS in NEAPP-AM treatment, NOS2 cells were pre-incubated with NAC or BSO, followed by the addition of NEAPP-AM for the indicated exposure time. The viability of NOS2 cells was assayed 24 hours after NEAPP-AM treatment using the cell viability assay. As shown in Fig. 3, the growth-inhibitory effects of NEAPP-AM-180 were completely inhibited on being pretreated with NAC, while the viability decreased by roughly 30% after NEAPP-AM-300 with NAC, indicating that there might be insufficient molecules of NAC to abolish ROS activity. The BSO with NEAPP-AM decreased cell viability under all treatment conditions; especially, BSO with NEAPP-AM-120 led to a significant decrease by 50%, compared to the control (NEAPP-AM treatment alone) (*P*<0.01). These results suggest that ROS in cells produced by NEAPP-AM play a critical role in anti-tumor effects against EOC cells.

### Effect of NEAPP-AM on chemo-resistant cell lines

**Figure 4:**
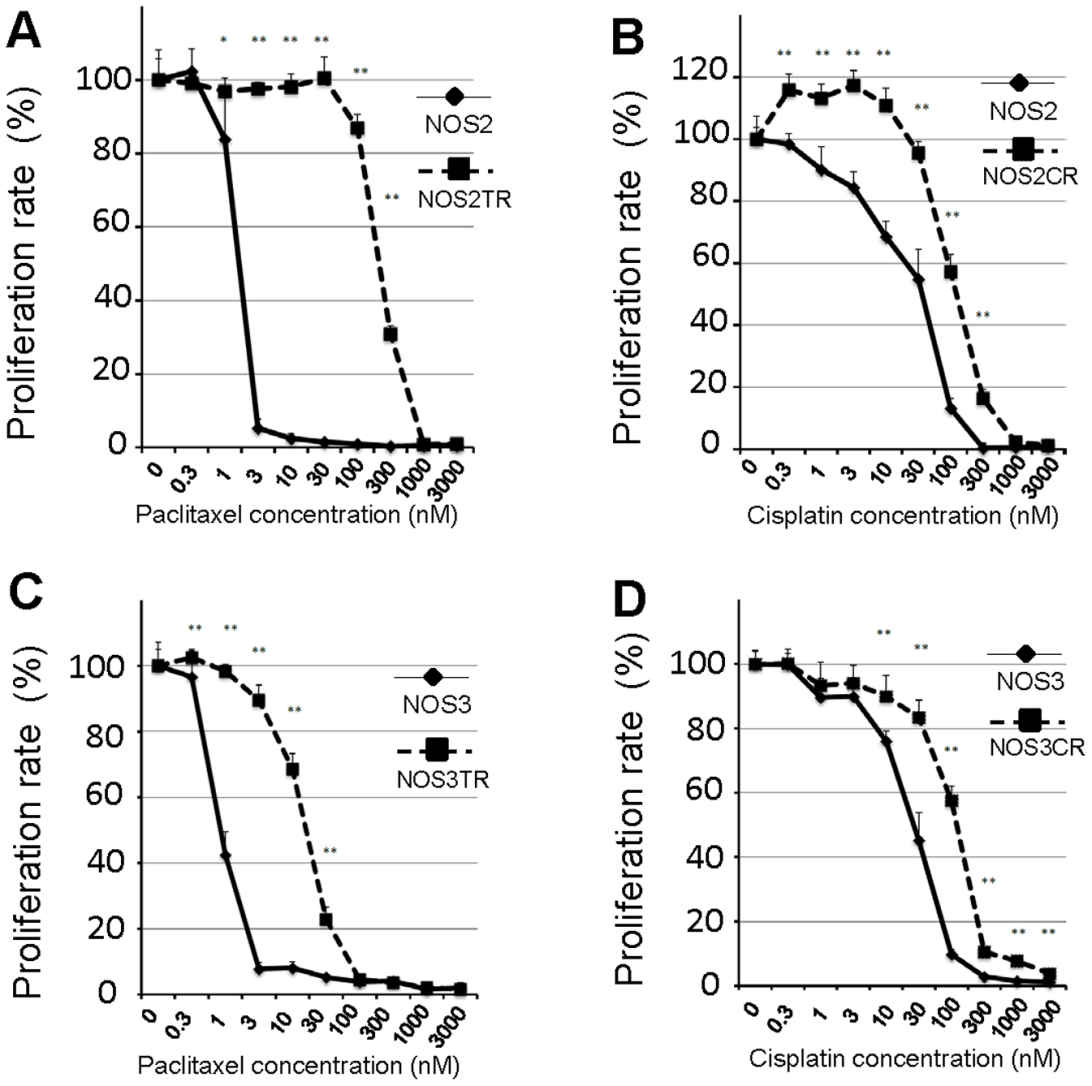
Chemo-sensitivity assay of parental and resistant cells. *A, B*: Paclitaxel-sensitivity assay in resistant NOS2TR (A) and NOSCR cells (B) compared to parental NOS2 cells. *C, D*: Cisplatin-sensitivity assay in resistant NOS3TR (C) and NOS3CR cells (D) compared to parental NOS2 cells. Each point represents the mean, and the bars show SD. Data are representative of at least three independent experiments. *P<0.05, **P<0.01 versus parental cells.

**Figure 5:**
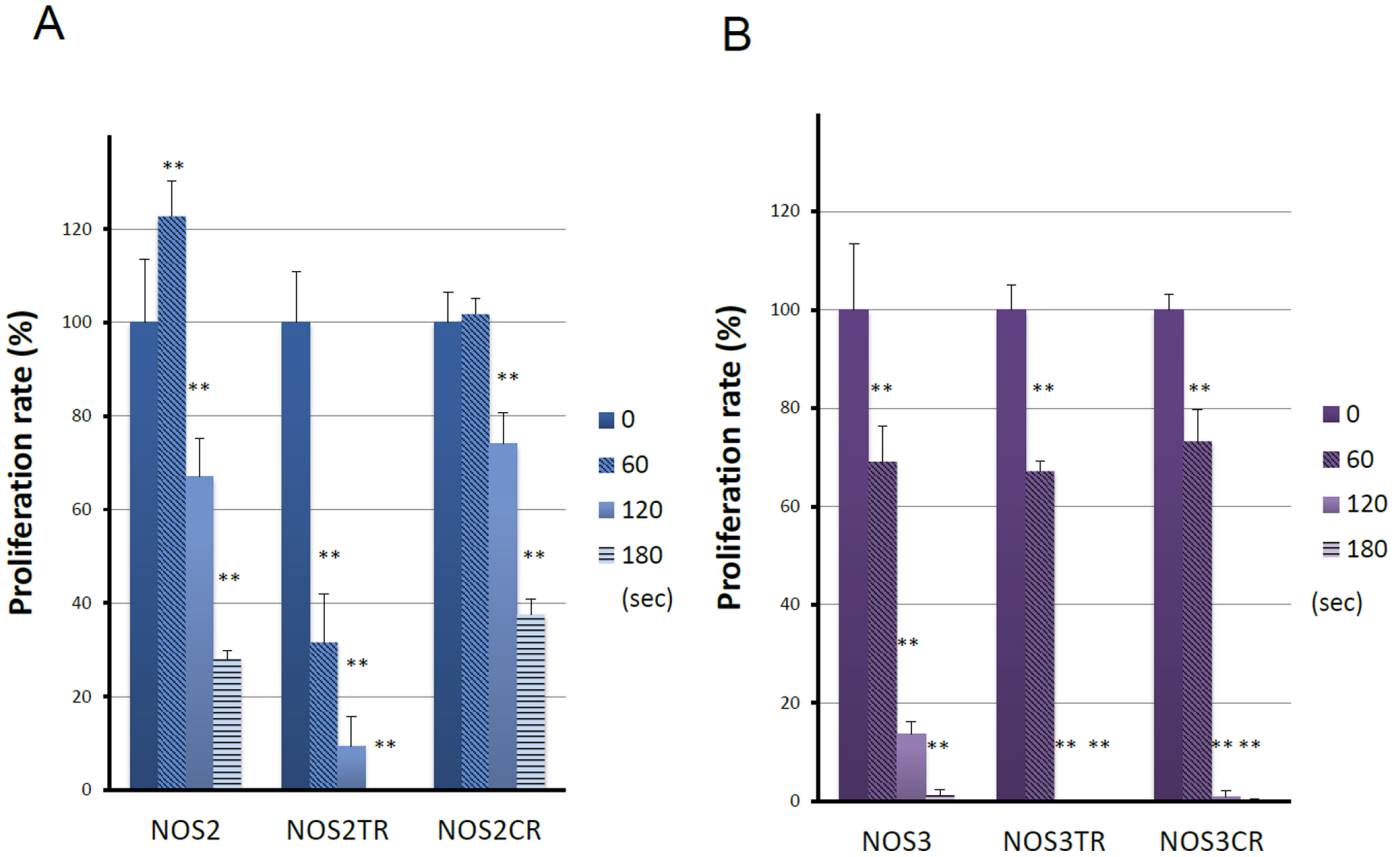
Effect of NEAPP-AM on chemo-resistant cell lines. Cell viability of NOS2 (A) NOS3 (B), parental cells and paclitaxel ^39^ or cisplatin(CR) resistant NOSTR and NOSCR cells treated with NEAPP-AM was measured by the cell viability assay. Results are presented as means ± SD of a representative of at least three independent experiments. *P<0.05, **P<0.01 as compared with the 0 sec control.

To assess whether NEAPP-AM treatment could affect chemo-resistant cell lines, we used paclitaxel and cisplatin-resistant cells generated by NOS2 and NOS3 cells (NOS2TR, NOS2CR, NOS3TR, and NOS3CR cells). We first confirmed the extent of resistance to paclitaxel or cisplatin of these cells. Figure 4 shows the paclitaxel or cisplatin-sensitivity assay of parental NOS2 and resistant NOS2TR cells. Marked chemoresistance to paclitaxel and cisplatin was noted in NOS2TR and NOS2CR cells, compared to parental NOS2 cells, respectively. The IC50 values of these cells were as follows: paclitaxel: 1.9/231.8 in NOS2/NOS2TR cells, cisplatin: 38.2/135.6 in NOS2/NOS2CR cells. Similar results were confirmed in NOS3TR and NOS3CR cells {IC50 value: paclitaxel: 0.9/18.2 in NOS3/NOS3TR cells, cisplatin: 38.2/132.2 in NOS3/NOS3CR cells}. As shown in Fig. 5A, cell viability was evaluated 24 hrs after NEAPP-AM treatment using the cell viability assay. Proliferation rates in both NOS2, and NOS2CR cells reduced to around 70% after NEAPP-AM-120 treatment. Interestingly, proliferation rate in NOS2TR reduced to 9% after NEAPP-AM-120 treatment, being more sensitive to NEAPP-AM. In addition, a similar tendency was noted in other NOS3TR and NOS3CR cells (Fig. 5B).

### A mechanism of NEAPP-AM-induced apoptosis on NOS2 and NOS2TR cells

**Figure 6:**
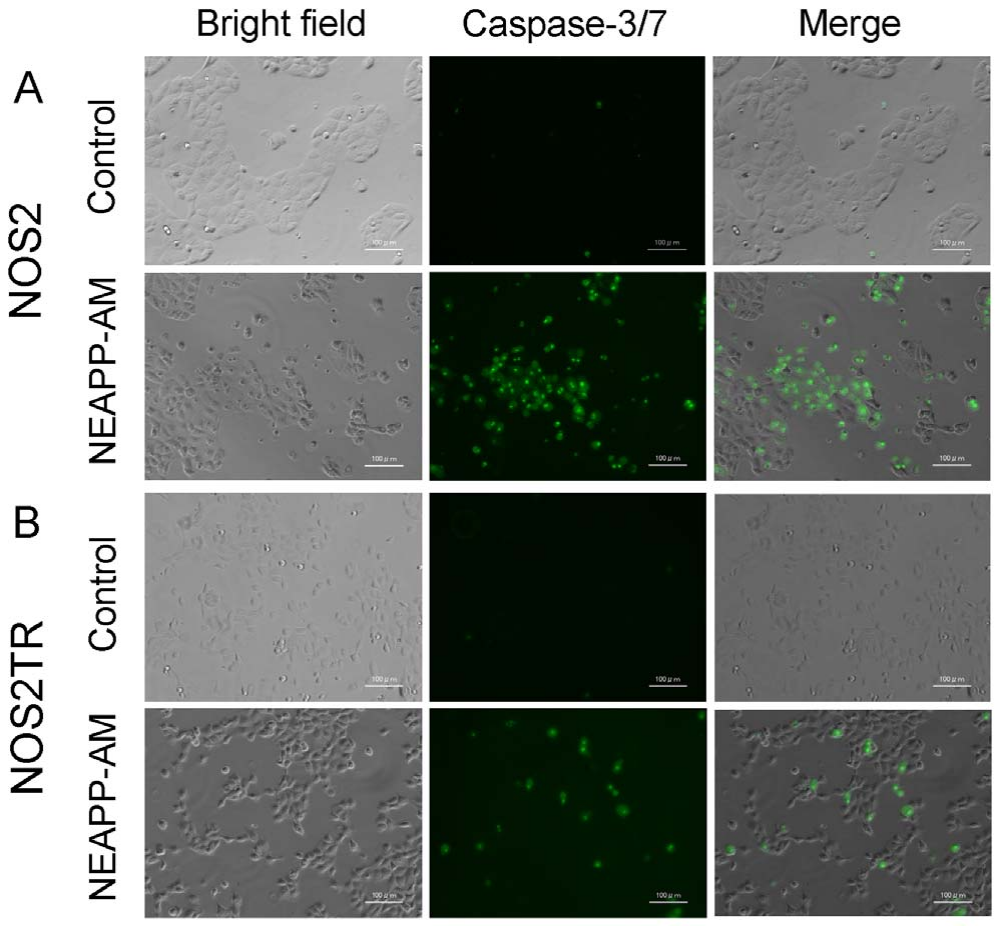
Morphological changes and images of caspase-3/7 activation and intracellular ROS detection in NOS2 and NOS2TR cells after NEAPP-AM treatment. The activity of caspase-3/7 was determined with the CellEvent™ caspase-3/7 Green Detection Reagent (Invitrogen) according to the manufacturer's instructions. Four hrs after NEAPP-AM treatment, NOS2 (A) and NOS2TR (B) were observed by microscopy and fluorescence microscopy. Morphological changes were evaluated under a microscope. There were shrinkage and blebbing cells treated with NEAPP-AM, indicating apoptosis induction. The scale bar corresponds to 100 µm.

In our previous study, direct NEAPP treatment induced cell apoptosis in EOCs. We subsequently assessed whether the cytotoxic effect of NEAPP-AM was associated with the induction of apoptosis. Caspase-3/7 activity was assayed at 4 hrs after NEAPP-AM treatment by loading the fluorescent substrate CellEvent™ caspase-3/7 Green Detection Reagent. As expected, compared with control cells, NEAPP-AM treatment caused morphological changes (shrinking, rounding up and detachment from dishes),which were typical of apoptosis, and the activation of caspase3/7 in both cell types (Fig. 6).

These results suggest that NEAPP-AM induced apoptosis through caspase-3/7 activation.

**Figure 7:**
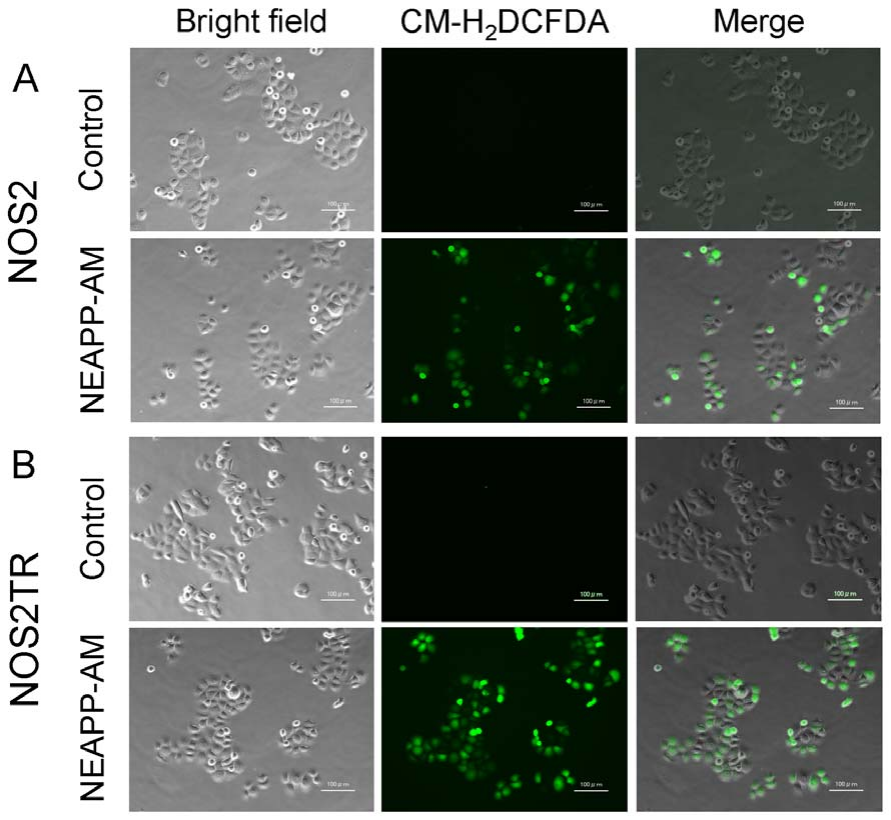
Intracellular ROS generation. *A, B*: ROS detection with or without NEAPP-AM treatment. NOS2 (A) and ROS2TR (B) cells treated with or without NEAPP-AM with preloading of CM-H_2_DCFDA. NOS2 (A) and NOS2TR (B) Cells were preloaded with CM-H_2_DCFDA for 15 min and treated with NEAPP-AM. Images were visualized using a fluorescence microscope. Representative images of cells from three independent experiments are shown. The scale bar corresponds to 100 µm.

Furthermore, it has been reported that NEAPP treatment up-regulates the production of intracellular ROS, leading to the apoptosis in cancer cells. In order to determine the role of ROS plays in NEAPP-AM induced apoptosis, we first examined the production of ROS using the oxidant-sensitive fluorescent probe CM-H_2_DCFDA. The results revealed that treatment with NEAPP-AM increased the intensity of the DCF signal compared with the control in both NOS2 and NOS2TR cells (Fig. 7).

### Growth-inhibitory effect of NEAPP-AM on tumor xenografts in mouse model

**Figure 8:**
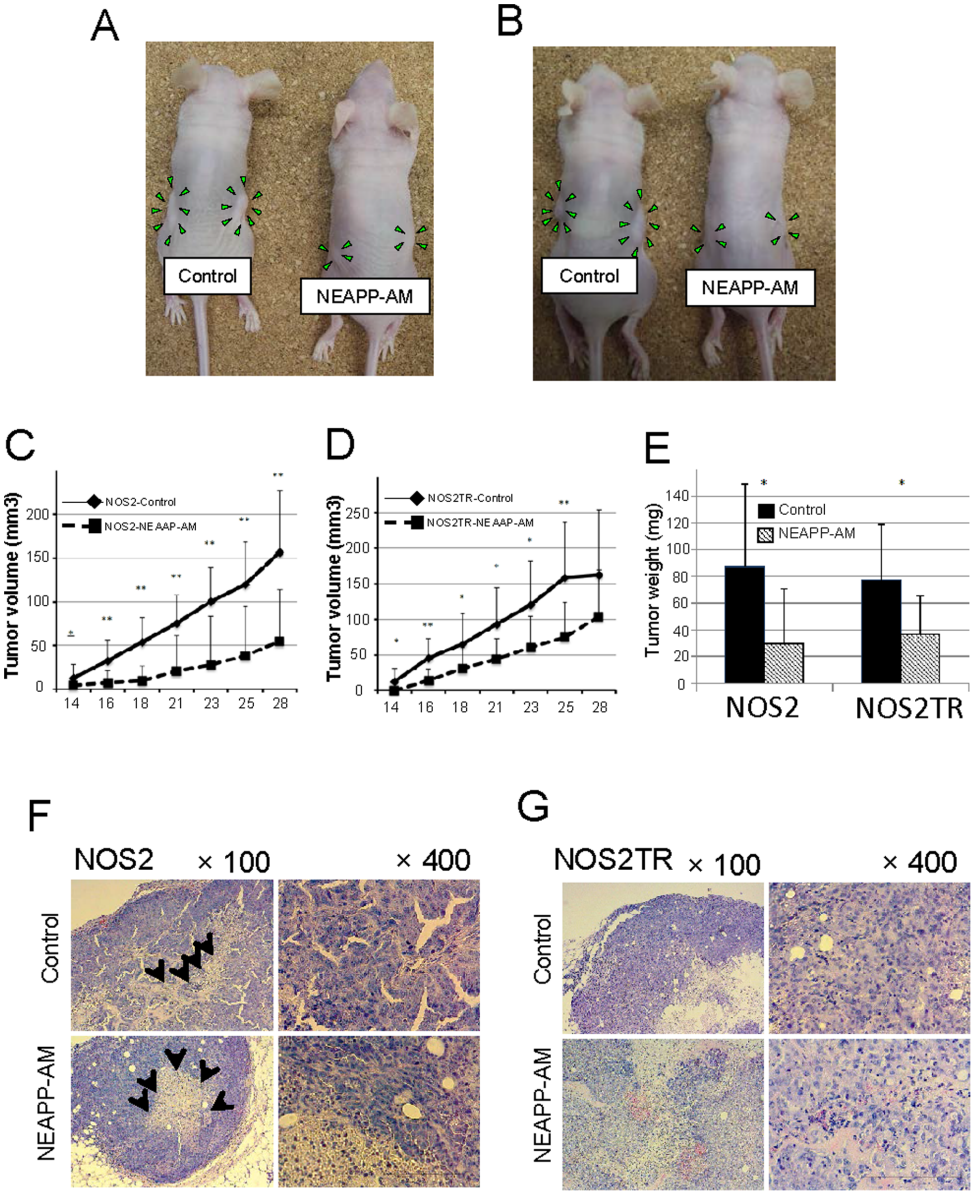
Anti-tumor effect of NEAPP-AM in mice with NOS2 and NOS2TR cell lines. *A, B*: The macroscopic observation of nude mice bearing subcutaneous NOS2 (A) and NOS2TR (B) tumors on both flanks. Mice were injected with NOS2 and NOS2TR cells and then received medium alone or NEAPP-AM. A total of 0.4 mL of medium or NEAPP-AM was administered locally into both sides of mice three times a week. All mice were sacrificed at 29 days after implantation. Green arrowheads indicate tumor formation. *C, D*: Time-dependent changes in the tumor volume in xenografted models are shown, medium alone (♦) or NEAPP-AM (▪). Each point on the line graph represents the mean tumor volume (mm^3^) for each group on a particular day after implantation, and the bars represent SD. *P<0.05, **P<0.01 versus control. *E*: The bar graph shows the mean tumor weight of the excised NOS2 and NOS2TR tumors from NEAPP-AM or control groups. P-values versus the control group were 0.017 and 0.014 in the NOS2 and NOS2TR groups respectively. *F, G:* Histological analysis of tumors. Representative images of hematoxylin and eosin staining of paraffin-embedded tissue sections from xenografted tumors implanted in NOS2 (F) and NOS2TR (G). Black arrows indicate necrotic core within the tumor. The scale bar corresponds to 100 µm.

We finally investigated the potential anti-tumor properties of NEAPP-AM in nude mice receiving subcutaneous xenografting. The experimental conditions of this *in vivo* model were set to assess whether NEAPP-AM could inhibit tumor formation due to microdissemination. Subcutaneous tumor formation was observed at least approximately 10 days after the inoculation of mice with NOS2 or NOS2TR cells. Subsequently, periodic s.c. treatment with NEAPP-AM was performed, as described in Materials and Methods. As shown in Fig. 8, the calculated tumor volumes on day 28 were 300±136 (mm^3^ ± SD) and 163±91 in the NOS2 and NOS2TR control groups and 55±60 and 104±66 in the NOS2 and NOS2TR groups treated with NEAPP-AM, respectively (p = 0.0014 and 0.097, respectively). NEAPP-AM injection resulted in an average inhibition of NOS2 cell-inoculated tumor weight by 66% (*P* = 0.017) and NOS2TR cell-inoculated tumors by 52% (*P* = 0.014), as compared with the control. Accordingly, NEAPP-AM significantly reduced the growth of both parental and chemo-resistant EOC tumors. During the experiment, we confirmed that the NEAPP-AM administration was nontoxic by observing mouse weights, survival, and behavior, and no complications such as anaphylaxis and skin necrosis were detected.

We also observed histological differences in the tumor sections between NEAPP-AM-treated and -untreated groups in both cell lines.

It should be noted that papillary growth, a clear character in ovarian serous adenocarcinoma from which NOS2 and NOS2TR were isolated, was detected. On the contrary, NEAPP-AM-treated tumors lost this characteristic. It was a similarity that both tumor groups had central necrosis. In subcutaneous tumors formed by the inoculation of NOS2TR cells, we noted the same histological appearance identical to serous adenocarcinoma and central necrosis although it was lesser extent to those of parental NOS2 cells. This indicated that NEAPP-AM inhibited tumor proliferation but there was no evidence to help elucidate the mechanisms of this phenomenon *in vivo*.

## Discussion

Numerous EOC patients respond well to platinum in combination with paclitaxel, which is the first choice for EOC. However, most of those patients develop recurrence and acquired resistance to various chemotherapeutic agents. Although such patients actually received repeated or other types of cytotoxic agent with markedly painful side effects, these treatments have not led to a satisfactory oncologic outcome. If such patients are effectively treated by alternative, less toxic therapy, they could be spared unnecessary painful symptoms.

In our earlier report, we demonstrated that direct irradiation of NEAPP significantly decreased proliferation rates of EOC cells compared to fibroblast cells [16]. This suggests that plasma may selectively kills EOC cells through the induction of apoptosis. However, considering the well-known characteristics of EOC, disseminating and implanting throughout the peritoneal cavity, intraperitoneal (IP) treatment may be more practical from a clinical aspect. If we applied the plasma as an IP treatment modality, a new therapeutic technology may be developed targeting intraperitoneal microscopic and/or macroscopic tumors, facilitating higher tumor penetration and accumulating cytotoxic effects in the peritoneal cavity. We recently confirmed that NEAPP-AM also exhibits a selective anti-tumor effect on glioblastoma cells (U251SP) but not normal human brain astrocytes (ACBRI-371) [17], assuming that NEAPP-AM would show low-level toxicity in a living system. However, there has been no report on the effect of plasma or plasma-activated medium on chemo-resistant cells despite the fact that plasma treatment has been reported to induce cell death in various cancer cells [5], [14], [15], [25]. Thus, in our current examination, we attempted to verify whether plasma also has an anti-tumor effect on chemo-resistant EOC, which was previously established [18], [19].

Several previous studies have demonstrated that plasma generates a large amount of ROS, leading to DNA damage and cell death [5], [14], [15], [25]. Indeed, our plasma system (NEAPP) has also been characterized in previous reports in which some ROS, such as hydroxyl radicals, singlet oxygen radicals, nitrogen oxide, and nitrogen, were detected in the plasma by OES [16]. Here, we used ‘nonequilibrium atmospheric pressure plasma-activated medium (NEAPP-AM)’; thus, we should note which reactive species (RS) in the liquid phase lead to the anti-tumor effects on EOCs. It has been reported that water exposed to plasma became acidic, in which hydrogen peroxide and nitric/nitrous acid were dissolved, leading to the inactivation of microbe [26]–[28]. Moreover, plasma-treated medium also down-regulated cell viability by RS and secondary products produced from materials in the medium, as demonstrated in previous reports [13], [15]. Nevertheless in the absence of an established NEAPP generator, various researchers have developed these devices. Therefore, it is difficult to simply compare the properties among plasma-generating systems. We should further investigate the mechanical standardization with the aim of future practical use.

Not only RS, but also UV, shock waves and an electric field are simultaneously produced with plasma. Therefore we were not able to ignore their direct plasma effects in our prior study [22], but might not need to consider them in the current study, focusing on the indirect effects of plasma.

Thus, we also demonstrated that incubation with NEAPP-AM led to an anti-proliferative effect in parallel with intracellular ROS up-regulation on chemo-resistant cells as well as parental cells, and NEAPP-AM with NAC treatment reversed the anti-tumor effects, suggesting that most of its effects are attributable to ROS from NEAPP. NAC has been widely used as an anti-oxidant and well investigated its reactivity for ROS. It has been reported that NAC directly scavenge hydroxyl radical, hydrogen peroxide and hypochlorous acid but not superoxide radical, suggesting the possibility that the anti-tumor effects on EOCs are attributed to at least one of them [29], [30]. Low levels of ROS are mitogenic and promote cell proliferation and survival [31]–[33]. In our analysis, with exposure for a shorter duration, cell proliferation was up-regulated by approximately 20%. Our results were consistent with this phenomenon.

Meanwhile, it has been documented that different cells have different redox-buffering systems to maintain intracellular redox homeostasis, once disruption of the balance leads to transient or permanent cell cycle arrest and induces cell apoptosis [34]–[36]. Under mild conditions of plasma exposed-medium down-regulating the cell viability through apoptosis, cells even showing chemoresistance have exhibited differential sensitivity to NEAPP-AMs, suggesting that NEAPP-AM is a new therapeutic strategy, showing selectivity, even in chemo-resistant cells. Moreover, our current data shows that the inhibition of glutathione synthesis by BSO enhanced the *in vitro* anti-tumor efficacy of NEAPP-AM. Accordingly, based on these results, the growth-inhibitory effect of NEAPP-AM also appears to be mediated through ROS generated by plasma, and it could be enhanced by the GSH inhibitor BSO. Indeed, several studies have reported the cytotoxic effect of ROS-generating agents on chemoresistant cells, which could be enhanced by the inhibition of glutathione synthesis, which plays an important role in the redox system, or they could sensitize cancer cells to chemotherapies [37], [38]. In the latest report, non-thermal plasma (NTP) in combination with chemotherapy, gemcitabine, synergistically inhibited human pancreatic cancer cell proliferation *in vitro* and *in vivo* using direct plasma gun [39]. However, there are no detailed experiments with respect to both plasma-activated medium and paclitaxel generate ROS in cancer cells by different mechanisms, so the combination of these two agents may be a synergic strategy to overcome chemoresistance. Hence, investigations on the effect of combination therapy are required in a future study.

The plasma-activated medium induced a significant inhibition of tumor growth in mice bearing both parental NOS2 and chemoresistant NOS2TR cells. However, this anti-tumor effect was not so marked compared to that of the *in vitro* study. Probably, a part of ROS generated by plasma is degenerated by a higher antioxidant capacity derived from intrinsic ROS scavengers in living organisms. Accordingly, how to manage ROS scavenger is a critical challenge to improve the therapeutic effect of indirect plasma treatment *in vivo*. Although we used a s.c. xenograft model in order to assess tumor growth easily, in this study, we confirmed the effect of NEAPP-AM against ovarian cancer cells *in vivo*. This is the first study to demonstrate that plasma-irradiated liquid can also inhibit the growth of malignant tumors *in vivo*. Moreover the effect against chemo-resistant cells was equivalent to that against chemo-sensitive cells by the weight of the resected tumors. Next, we are considering applying plasma-irradiated liquid I.P. therapy to a murine peritoneal metastasis model with EOCs and its chemo-resistant cells in the presence or absence of antineoplastic agents and/or ROS scavenger inhibitors. This treatment, with its lower-level adverse effects due to its selectivity for cancerous cells [16], [17], may be an excellent alternative treatment for chemo-resistant peritoneal metastasis, one of the main obstacles to ovarian cancer treatment. We did not demonstrate the differences of the anti-proliferative efficiency between the direct plasma and indirect plasma, NEAPP-AM. The major mechanisms of the anti-tumor effects in both situations of plasma irradiation was likely to be the same, if it was induced by dissolved ROS in medium, in which there was a homogeneous generation of ROS due to the convective flow by gas or sufficient immixture before the exposure to cells. However, considering the intraperitoneal treatment for numerous micrometastatic disseminations of EOCs, direct plasma irradiation therapy cannot target all the numerous tumors throughout the peritoneal cavity. In this context, NEAPP-AM would be more easily put into practice since it is thought that aqueous ROS covers them via peritoneal fluid.

In conclusion, we demonstrated that plasma-activated medium also had an anti-tumor effect on chemo-resistant cells *in vitro* and *in vivo*. To apply the plasma treatment for advanced or recurrent EOC, we suggest adopting indirect plasma therapy instead of direct plasma considering intraperitoneal administration in the future. The indirect plasma therapy is a promising treatment option for EOC and will contribute to a better patient prognosis in the future.
